# Effects of Age and Expertise on Mental Representation of the Throwing Movement Among 6- to 16-Year-Olds

**DOI:** 10.3389/fpsyg.2022.799316

**Published:** 2022-04-12

**Authors:** Michael Gromeier, Thomas Schack, Dirk Koester

**Affiliations:** ^1^Neurocognition and Action – Biomechanics Research Group, Bielefeld University, Bielefeld, Germany; ^2^Center of Excellence “Cognitive Interaction Technology” (CITEC), Bielefeld University, Bielefeld, Germany; ^3^Research Institute for Cognition and Robotics (CoR-Lab), Bielefeld University, Bielefeld, Germany; ^4^BSP Business School Berlin, Berlin, Germany

**Keywords:** mental representation, SDA-M, motor skill development, movement expertise, throwing movement, overarm throwing motion, cognitive performance

## Abstract

The aim of this article was to assess the development of mental representation of the overhead throwing movement as a function of age and expertise. The mental representational structure of the overhead throwing movement was measured using the Structural Dimensional Analysis-Motoric (SDA-M) method that reflects the organization of basic action concepts (BACs). BACs are fundamental building blocks of mental representations, which comprise functional, sensory, spatiotemporal, and biomechanical characteristics of a movement (Schack, 2010). In this study, novices and handball athletes (*N* = 199) each were grouped according to the level of development in motor ontogenesis (in childhood, pubescence, and adolescents). Male and female handball athletes played in the highest leagues of their age groups. As a result, novices of all age groups showed the same unstructured mental representation. Athletes in the earliest age band resemble all novices’ groups and showed similar unstructured mental representation, whereas athletes within pubescence and adolescents showed functionally well-structured representations, which were similar to the structure of the reference group (*N* = 8). These results are consistent with a previous investigation of related quantitative and qualitative performance parameters of the overhead throwing movement (Gromeier et al., 2017). Without an increased training, neither the throwing performance nor the associated mental representation is unlikely to improve further by itself or automatically.

## Introduction

The overhead throwing movement is a complex and basic motor skill and a fundamental requirement for a number of sports, such as handball, baseball, and javelin. Typical handball-specific situations in which the overarm throwing movement comes into effect are 7 m throws, free throws, backcourt throws, and the initiation of fast breaks (Kolodziej, 2010). This motor skill is an important part of school and university education, as well as belonging to the club sport. The overhead throwing movement is a highly demanding coordinative skill, and it is said to be “one of the most difficult fundamental motor skill[s] for children and adults and its acquisition requires coordination of the whole body” (Hamilton and Tate, 2002, p. 49). During learning of complex motor skills, children and adolescents are repeatedly confronted with excessive demands of coordination. Due to the complexity of the overhead throwing movement, difficulties in movement performance can often be observed in exercises and game situations (e.g., physical education, university education, or club sports).

### The Overhead Throwing Movement

A large number of studies have examined expertise and gender-related differences in the overhead throwing movement in different handball and throwing situations (e.g., Hicks, 1931; Wild, 1938; van den Tillaar and Ettema, 2004; Laffaye et al., 2012; Karadenizlil et al., 2014; Rousanoglou et al., 2015; Maselli et al., 2019) and a variety of disciplines (Lombardo and Deaner, 2018). Significant differences in throwing velocity and throwing range were mainly demonstrated in favor of men (Keogh, 1969; Morris et al., 1982; Thomas and French, 1985; Geese, 1997; Roberton and Konczak, 2001; Zhu et al., 2009). Other studies recognized differences in throwing accuracy (Keogh, 1969; Vogt, 1978; Thomas and French, 1985; Geese, 1997; Gaschler, 1998; Ahnert, 2005) and in the qualitative characteristics of overhead throwing movements and typically, were also in favor of men compared with women (Keogh, 1969; Vogt, 1978; Thomas and French, 1985; Nelson et al., 1991; Geese, 1997; Gaschler, 1998; Ahnert, 2005; but see Gromeier et al., 2017).

Differences among novices, beginners, advanced, and experts were unambiguously demonstrated in throwing velocity and throwing accuracy (e.g., van den Tillaar and Ettema, 2004; Gorostiaga et al., 2005; Rivilla-García et al., 2010; Rousanoglou et al., 2015).

The studies of Gorostiaga et al. (2005) clarify quantitative differences between two handball male teams, namely, expert team, one of the world’s leading teams, and amateur team, playing in the Spanish National Second Division. Experts showed significantly higher velocity of throwing without stepping and three-step rhythm than amateurs using the same approach. Rousanoglou et al. (2015) found similar results when comparing between experts and amateurs. Amateurs performed with significantly lower values in velocity of throwing and worse throwing accuracy. Rivilla-García et al. (2010) found significant differences in velocity between senior players and U-18 players in different throwing situations. Senior players were found to perform significantly better than the U-18 players in all four throwing situations. The quantitative characteristics of throwing movements of experts and beginners in handball were also analyzed by van den Tillaar and Ettema (2004). They evaluated Norwegian handball players at the age of 20–24 years and found gender differences in the throwing velocity in favor of men compared with women.

In previous investigations, differences in quantitative and qualitative throwing performances of novices and athletes across a life span of 6–16 years of age were examined (Gromeier et al., 2017, 2019). It was shown that novices during childhood, pubescence, and adolescents and athletes in childhood have similar results in throwing performance of the overhead throwing movement. These results are different to the athletes in pubescence and adolescents. Among the athletes, a developmental spurt between the stage of childhood and the pubescence can be assumed. This is suggested by the significant improvements in the quantitative and qualitative movement performances. This confirms the results of Rivilla-García et al. (2010), Laffaye et al. (2012) and Rousanoglou et al. (2015), who showed that amateurs have significantly worse quantitative performance in throwing accuracy than expert players. The specific training among the athletes apparently leads to increasing quantitative and qualitative throwing performances. The outcome of novices differs drastically. No reliable improvement was observed among the novices. Throughout the 10-year period, novices did not increase their quantitative performance or their qualitative performance. These results contradict the studies by Roberton and Langendorfer (1980), Halverson et al. (1982), Roberton and Konczak (2001), and Goodway and Lorson (2008), who found age-related developments in throwing performances. Most of our fundamental motor skills, like overhead throwing movements are naturally acquired, as children play and move around by themselves. The innate motor and perceptual capabilities provide a basis for the acquisition of these patterns (Barela, 2013; Gromeier et al., 2017). The overarm throwing movement is a complex movement with a specific and clearly identifiable start and end. The movement can be divided into distinct phases (Werner et al., 1993). This type of throwing movement seems to follow a specific movement pattern determined by a proximal to distal sequence of segments. According to Wagner et al. (2014, p. 345), the “equal order of the proximal-to-distal sequencing and similar angles in the acceleration phase suggests there is a general motor pattern in overarm movements.” Also, and more importantly, there are specific and typically mistakes in the performance of the throwing movement characteristics including, stepping, backswing, humerus oblique under shoulder, no stepping, backswing, and trunk forward-flexion (Roberton and Langendorfer, 1980; Halverson et al., 1982; Roberton and Konczak, 2001; Goodway and Lorson, 2008; Gromeier et al., 2017).

The way of how these moving patterns will change over the time depends on training intervention, learning, and adaptation that will arise through perceptual-motor experiences that children would have during childhood (Barela, 2013; Gromeier et al., 2017, 2019). These results suggest that most of children and adolescents do not master skill performance and would not acquire the most proficient performance in the fundamental motor skills, like overhead throwing movements (Gallahue, 1982). The performance requirements for overhead throwing movement execution may be representing a natural limitation, caused among other things by the change in the living world. The difficulties in the performance of overhead throwing movement could be a possible hint of a motor skill “proficiency barrier” (Seefeldt, 1980). Gallahue (1982) argued that fundamental motor skills would require regular physical education, composed by structured practice, specialist teachers, and specific conditions, and promote gross motor development of children.

### Developmental Psychology in Youth Sport

Based on Piaget’s (1950) model of cognitive development and Erikson’s (1950) stages of psychosocial development, there are distinct developmental phases. In accordance with Piaget (1950) and Erikson (1950), Meinel and Schnabel (1998) described a phased development of movements and interpreted the respective stages of development as integration at a higher level of performance. Covering the entire life span, Meinel and Schnabel (1998) focused exclusively on sports motor aspects, which are similar to the development model of Gallahue et al. (2012). Gallahue et al. (2012) and Meinel and Schnabel (1998) focused on the phases of motor development and provided a solid introduction to the biological, affective, cognitive, and behavioral aspects within each developmental stage and differed mainly in the temporal organization of developmental stages.

However, there is likely to be certain variation in the physical, cognitive, social, and emotional development within each developmental stages, and each child develops at her or his own natural speed, and variety of motor and cognitive development is shown to proceed in a specific, predictable sequence, and a number of features of normal motor and cognitive development can be distinguished and improved as the children’s ages increased. The findings of De Waelle et al. (2021) indicate that age-related improvements of perceptual-cognitive skills are evident. The authors examined the development of anticipation, decision-making, and pattern recall in female volleyball players of six different age groups and find out that adolescents and adult athletes had superior accuracy and shorter response times than younger athletes. A study by Caeyenberghs et al. (2009) shows age-related changes of motor imagery in children using a cross-sectional design, with significant improvements of motor imagery occurring after 7–8 years of age. Related to comparable age bands, these results were underlined by Souto et al. (2020) who found out that motor imagery ability improved as a function of age. Overall, phases of motor and cognitive development can be seen as general guide to cognitive development and as sources of constraints on what structures and functions are available to the developing mind (Feldman, 2004).

Mental representations play a central role in the control and organization of action. Complex actions are believed to consist of hierarchical taxonomies comprising basic action concepts (BACs). BACs are cognitive clusters of (anticipated) movement effects and, therefore, contain integrated feature-based units that represent functional, sensory and spatiotemporal, and biomechanical characteristics of a movement (Schack, 2010). BACs combine functional movement features and provide the representational basis for the control of complex actions (Schack, 2004; Schack and Mechsner, 2006; Bläsing et al., 2009). Motor learning, and more precisely, learning by repeated execution of complex action, is reflected by functional changes in the relations and the groupings of BACs (e.g., Schack and Mechsner, 2006; Schack and Ritter, 2013). Consequently, motor performance becomes optimized, which may lead to automaticity of movement execution. Thus, well-structured patterns in mental representation are essential for the movement control (Schack, 2010). The storage of mental representation of overhead throwing movements can be evaluated using the Structural Dimension Analysis-Motoric (SDA-M; Schack, 2004). The SDA-M was developed based on the approach of the cognitive architecture of human motion (Lander and Lange, 1996; Schack, 2004, 2012). The SDA-M is a measuring method that objectifies the mental representations of movements and their internal structure in long-term memory by a successive splitting procedure (Schack, 2012).

Studies demonstrate that well-trained athletes and experts in various sports can be characterized by well-structured patterns of perceptual cognitive concepts, which is reflecting the functional phases of the motor skills. In contrast, novices show usually unstructured patterns in mental representation of motor skills (e.g., Schack and Mechsner, 2006; Bläsing et al., 2009; Stoeckel and Wang, 2011) and at early phases of the learning process of motor skills (e.g., Frank et al., 2016). Stoeckel and Wang (2011) analyzed the anticipatory motor planning and the development of mental representation of grasp posture of children. School children aged 7, 8, and 9 years participated in that study (Stoeckel and Wang, 2011). The mental representation was evaluated using a splitting procedure. The children successively judged the similarity of pictures depicting an object that was grasped in a comfortable or uncomfortable manner. The results highlight that 9-year-old children show clear cluster solutions in the representation structure. They distinguished uncomfortable grasp postures from comfortable grasp postures. The mental representations of 7- and 8-year-old children were not clearly structured in uncomfortable grasp postures and comfortable grasp postures. These results highlight the development of mental representation of motor skills during childhood as a function of age.

Among the key issue is how structured mental representations can arise during motor skill acquisition in throwing in schools and club sports. This study analyzed mental representations of overhead throwing movement among 6- to 16-year-olds to find out whether an overhead throwing movement improvement is accompanied by cognitive improvement.

The aim of this study was to examine the mental representation of the overhead throwing movement of different age groups and expertise. We expected that a growing expertise’s improvement in quantitative and qualitative throwing performance is accompanied by an improvement of the mental representation (Frank et al., 2016). Among athletes, it is hypothesized that with an increasing training level, children would distinguish technically correct and technically faulty clusters of movement representations. Moreover, at a certain level, athletes are expected to show similar cluster solutions in line with the reference representation structure. It is also hypothesized that novices distinguish technically correct and technically faulty BACs with an increasing age, but compared with athletes, they show less growth. Thus, we predict that the mental representation of overhead throwing movements would develop as a function of age concerning novices and as a function of expertise concerning athletes (Stoeckel and Wang, 2011; Frank et al., 2013, 2014, 2016).

## Materials and Methods

### Participants

A total of 206 participants, aged between 6 and 16 years (*M*_age_ = 11.31 years; SD_age_ = 2.989), 89 women and 117 men, took part in the study. The group of athletes (*N* = 96, *M*_age_ = 11.93 years; SD_age_ = 3.190) consisted of 37 female and 59 male subjects. All of these experts played active handball in the highest league of their age group. The sport clubs of these teams have a competitive sport orientation with a greater exercise frequency and intensity, thus enhancing practice and training. Moreover, athletes with competitive sport orientation often are highly motivated and trying to seek achievements in sport. The group of novices (*N* = 110, *M*_age_ = 10.76 years; SD_age_ = 2.702) consisted of 52 women and 58 men and came from public comprehensive schools. These participants had no prior experience in sports such as handball, baseball, tennis, volleyball or track, and field neither in club sports nor leisure groups. Thus, movement experiences in throwing and throwing skills have mainly developed by the private sports in the family, leisure time, and school education. Children and adolescents that have been active in other sports (e.g., dancing, gymnastics, martial arts, and swimming) were included in the group of novices.

The cluster solutions of the tested subjects were compared with those of reference group (RG), which consisted of eight experts (*M*_age_ = 18 years; SD = 0.000), four women and four men. They were playing in the national handball Bundesliga-A-juniors and were partly in an enlarged cadre of senior Bundesliga-team (mean age of the training = 11.5 years, training frequency per week = 12.5 h). The investigated movement is highly relevant for throwing in handball athletes. In line with the study by Wiener (2011), it is said that typical handball-specific situations in which the overarm throwing movement comes into effect are 7 m throws, free throws, backcourt throws, and the initiation of fast breaks. Because of repetitive throwing movements in game and training situations, it is suggested that the overhead throwing movement is well established in motor skills of athletes. From a functional perspective, the main function phase is directly related to the main movement goal, and it is identical to main functional phase of various kinds of throwing, which are used in real-game situations, e.g., jump throw or throwing with run-up (Göhner, 1979). Therefore, the training concept of the German Handball Association provides binding guidelines regarding the use the overhead throwing movement in 7-m throws (DHB, 2013). Due to repetitive throwing movements in game and training situations, it is suggested that the overhead throwing movement is well established in motor skills of athletes. To take part in the experiment, the parents of the participants gave informed consent. Throughout the motor testing, all participants were healthy and in good condition. The study was performed in accordance with the ethical standards of the Declaration of Helsinki 1975 (World Medical Association [WMA], 2013). Our study was also approved by the ethics committee (“Ethik-Kommission”) at Bielefeld University and adhered to the ethical standards of the latest revision of the Declaration of Helsinki. For the data analysis, we grouped three age groups for two different skill levels according to the level of development in motor ontogenesis of Meinel and Schnabel (1998) (i.e., childhood, up to 12 years, *N* = 75 novices and *N* = 45 athletes; pubescence, above 12–14 years, *N* = 17 novices and *N* = 24 athletes; and adolescents, above 14–16 years, *N* = 18 novices and *N* = 20 athletes).

### Mental Representation Structure of the Overhead Throwing Movement

#### Apparatus and Stimulus

The stimuli were 10 pictures of the overhead throwing movement representing the respective BACs (see Table 1). Based on a pilot study with 15 students and expert’s surveys with two former national handball athletes, five of the BACs were always categorized as showing technically correct movements, and in contrast, five were categorized as showing technically faulty movements. Therefore, the pictures (BACs) can be divided into two categories. The faulty BACs showed common main faults in the movement, which have been described and proven by many authors (e.g. Roberton and Konczak, 2001; van den Tillaar and Ettema, 2004; Ahnert, 2005; Goodway and Lorson, 2008; Gromeier et al., 2017, 2019). The correct BAC in the other category showed the correct movement execution.

**TABLE 1:** The images used represent the BACs of the overhead throwing movement.

Technically correct movement	Technically faulty movement
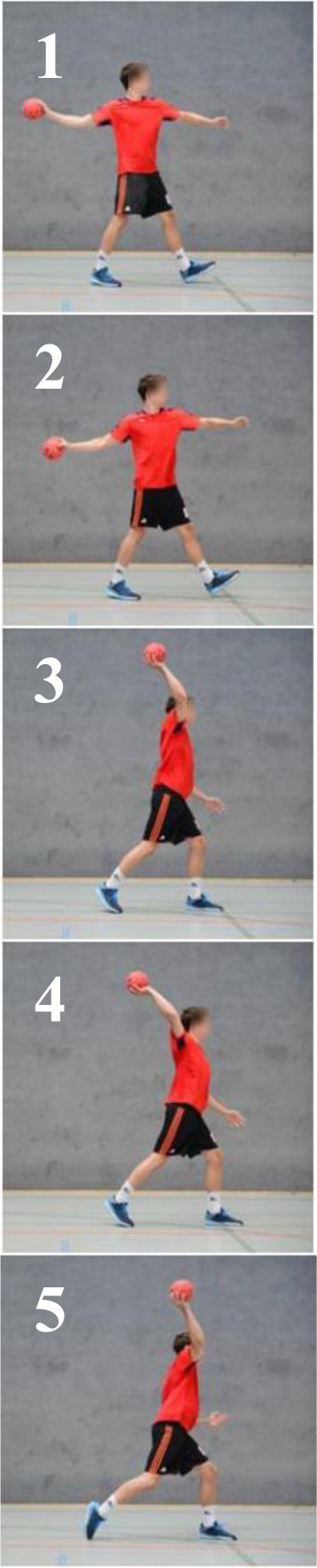	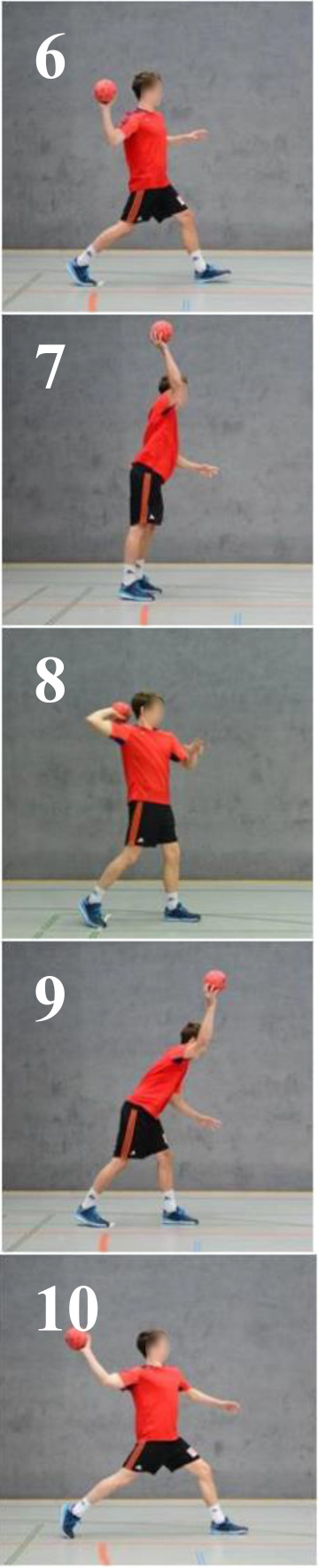

*The images were used in the SAD-M task. Images 1–5 present technically correct movements. Images 6–10 present typical errors in the overhead throwing movement. The picture number marks the BAC in the dendrogram. Concepts: 1–2 backswing; 3–5 throwing motion; 6 humerus oblique under shoulder; 7 no stepping; 8 backswing; 9 trunk forward-flexion; and 10 big step.*

The stimuli had a size of 9.0 cm × 9.0 cm (250 × 250 pixels) and were shown on a laptop (Sony Vaio). The participants sat approximately 50 cm away from a 15-inch laptop screen.

#### Task and Procedure

The mental representation was assessed using the SDA-M (Schack, 2004). For this, we determined the distances of the mental link of all relevant BACs by a splitting technique. The participants replied verbally and judged the similarity of the BACs with one another. Therefore, each picture (*N* = 10) was presented as an anchor. This anchor was displayed in the upper half of the screen. The remaining pictures were successively displayed below them and were classified to the anchor picture as similar or not similar. The participants were individually tested in a separate room, without any time pressure. Depending on the age of the participants, the testing took up to 25 min. The participants could cancel the experiment at any time without having to provide reason.

The participants were instructed to classify from their own perception, whether the two pictures shown at the laptop screen were similar or not. In case of similarity, both depicted movements should be perceived as fluent and pleasant respectively comfortable, and they show an appropriate solution of the movement goal. Or both depicted movements should be perceived as non-fluent and unpleasant respectively uncomfortable, and they show an inappropriate solution of the movement goal (Stoeckel et al., 2012). If the pictures did not correspond, they were classified as dissimilar. According to this question, it was found out that in the RG, the technically correct pictures could be characterized as a clearly comfortable. In contrast, the faulty patterns for the RG are seen as explicitly uncomfortable (see Figure 2). The group analysis of the RG clarified a definite assignment of technically correct and technically faulty BACs, separated into clusters.

**FIGURE 1:**
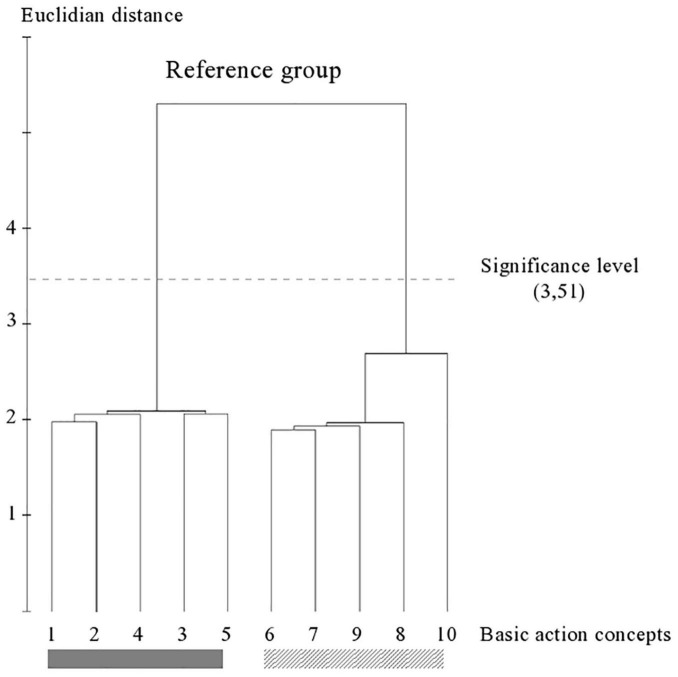
Outcome of the hierarchical cluster analysis for the reference group (*N* = 8, *M*_age_ = 18 years) displayed as a dendrogram (*p* = 5%; *d*_crit_ = 3.51). The basic action concepts (BACs) are marked with numbers below the dendrogram. The resulting clusters are highlighted with the gray solid bar (pictures 1–5, technically correct BACs) and diagonally striped bar (pictures 6–10, faulty BACs).

**FIGURE 2:**
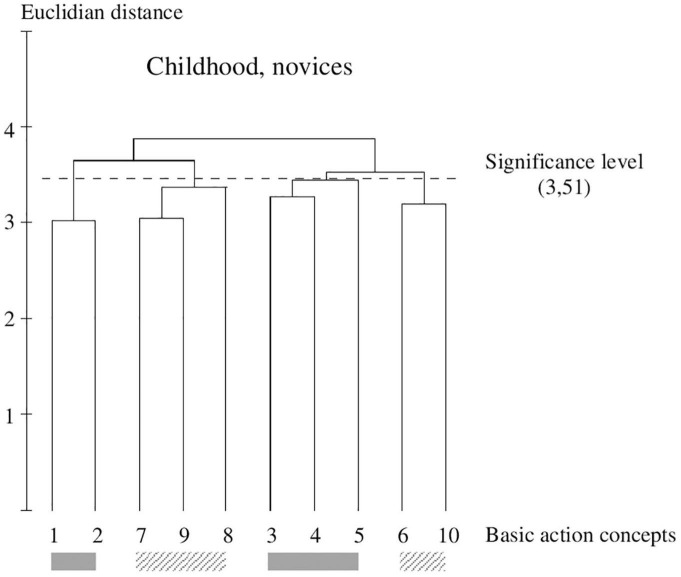
Outcome of the hierarchical cluster analysis of novices within childhood (*N* = 75) displayed as a dendrogram. The dashed line clarifies the critical value of *d*_crit_ = 3.51, which corresponds to a significance level of 5%. The BACs are marked with numbers among the dendrogram and highlighted with the gray solid bar (technically correct BAC) and diagonally striped bar (faulty BAC).

Especially among the younger participants, it was necessary to explain the task clearly and understandably. For this matter, the test started with the following text (translation of German original):

*During the motor test you had thrown at a target with a ball. And maybe you remember games or situations in which you have thrown a ball or a snowball for example. I‘ll show you some pictures. On these pictures you can see a person, named Yannik throwing a ball, like you did. Yannik threw in two different ways. Sometimes he threw in a comfortable manner, and sometimes he threw in an uncomfortable manner. I want you to tell me, whether both displayed movements are comfortable or uncomfortable, or whether one displayed movement is comfortable and the other displayed movement is uncomfortable.*

#### Data Analysis

The experimental procedure of the SDA-M includes four steps. In the first step of the SDA-M, the distance between all images (i.e., BACs) by a multiple sorting task was analyzed. In a pairwise comparison, the participants assessed the similarity of all BACs with one another. In this splitting procedure, each concept was displayed as an anchor concept on a computer screen. Any remaining concepts were compared with that anchor successively. The participants were to decide whether the displayed concepts were related to each other. All concepts were compared with all other concepts. In the second step, the individual cluster solutions were formed by means of a hierarchical cluster analysis. As a result, an individual cluster with ten decision trees was formed and was depicted as a dendrogram (see Figure 2). Based on a significance level of *p* = 0.05, a Euclidean distance (*d*_crit_ = 3.41) was defined (cf. Schack, 2012, 2004). All connection points below the Euclidean distance *d*_crit_ were viewed as not significantly associated, while the connections points above Euclidean distance *d*_crit_ were viewed as significantly associated. In this study, an optimal dendrogram shows a two-cluster solution (Schack, 2012, p. 206); one cluster with technically correct BACs and one cluster with technically faulty BACs. The third step contains the measurement of invariance of cluster solutions within and between groups. In a final step, the authors assessed the structural invariance within and between group cluster solutions by means of an invariance measure (λ). The final comparison of λ with λ_crit_ = 0.68 makes statements about the manner in which the cluster solutions have significantly similar structure to a predefined reference (λ_*ideal*_ = 1). This means that there was a significant difference between clusters if λ < λ_crit_ = 0.68. If λ is greater than λ_crit_, there is a significant similarity, which corresponds to an alpha level of 5% (Schack, 2012). The Rand Index was used to compare the differences of the representation structure of age and gender groups.

The cluster analysis (Figure 1) shows the group structure of the RG, which represents the optimal cluster solution of the SDA-M. The horizontal dashed line indicates the critical value (*d*_crit_ = 3.51). The dendrogram of the RG shows a clear separation in technically correct (pictures 1–5) and faulty BACs (pictures 6–10). The hubs are highly interconnected and are clearly below the critical distance (*d*_crit_ = 3.51). Within the RG, it is possible to determine a definite structure by a significant separation in technically correct BACs underlined with a gray solid bar and faulty BACs underlined with a diagonally striped bar. If the value is λ_crit_ = 0.68 is reached or exceeded, the cluster solutions are invariant. Within the RG, the λ*-*values were between 0.71 and 1.00. Therefore, within the reference, the individual cluster solutions are identical.

## Results

**FIGURE 3:**
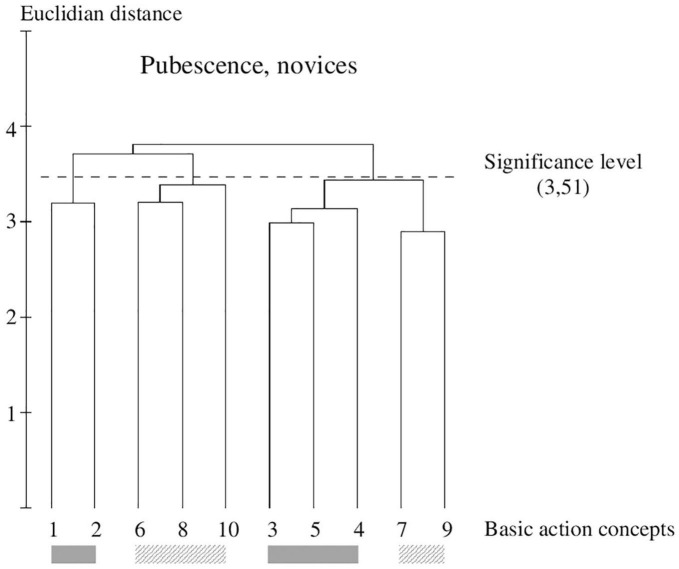
Outcome of the hierarchical cluster analysis of novices within pubescence (*N* = 17) displayed as a dendrogram. The dashed line clarifies the critical value of *d*_crit_ = 3.51, which corresponds to a significance level of 5%. The BACs are marked with numbers among dendrogram and highlighted with the gray solid bar (technically correct BAC) and diagonally striped bar (faulty BAC).

**FIGURE 4:**
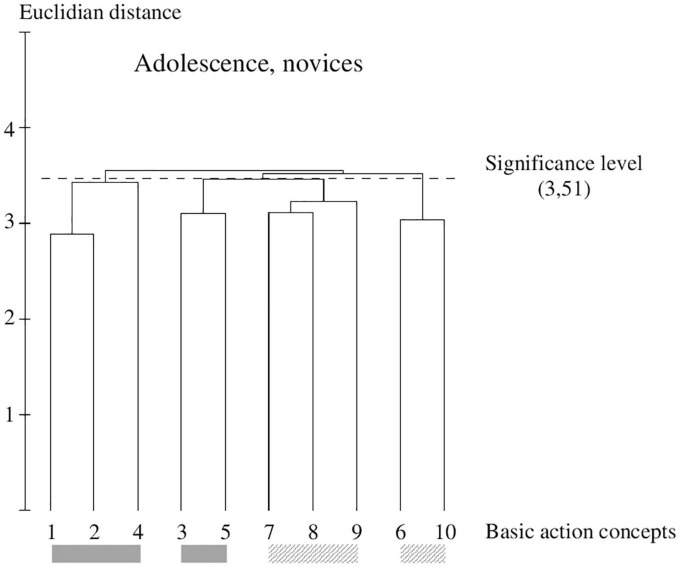
Outcome of the hierarchical cluster analysis of novices within adolescents (*N* = 18) displayed as a dendrogram. The dashed line clarifies the critical value of *d*_crit_ = 3.51, which corresponds to a significance level of 5%. The BACs are marked with numbers among the dendrogram and highlighted with the gray solid bar (technically correct BAC) and diagonally striped bar (faulty BAC).

**FIGURE 5:**
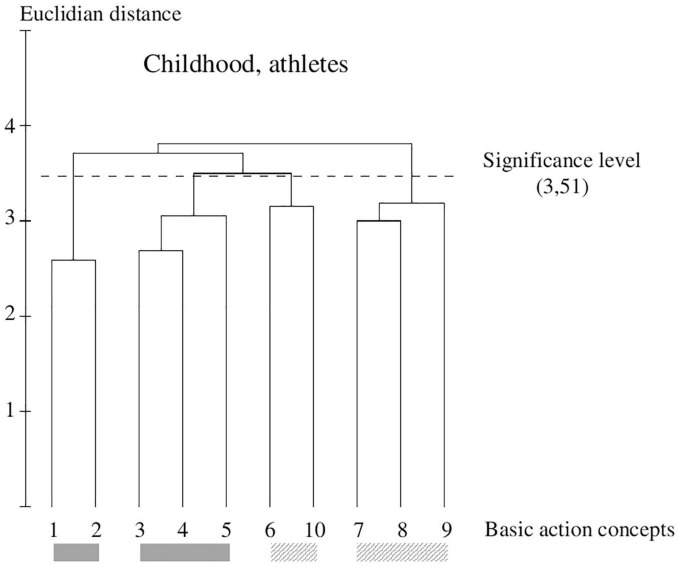
Outcome of the hierarchical cluster analysis of athletes within childhood (*N* = 45) displayed as a dendrogram. The dashed line clarifies the critical value of *d*_crit_ = 3.51, which corresponds to a significance level of 5%. The BACs are marked with numbers among a dendrogram and highlighted with the gray solid bar (technically correct BAC) and diagonally striped bar (faulty BAC).

**FIGURE 6:**
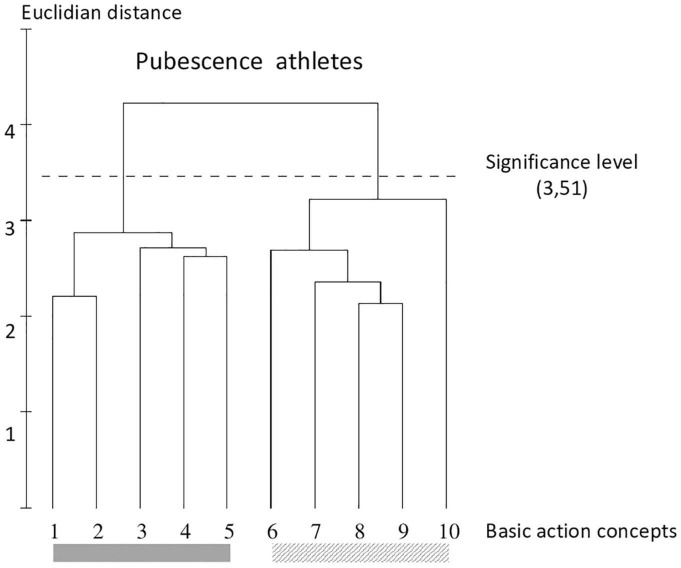
Outcome of the hierarchical cluster analysis of athletes within pubescence (*N* = 24) displayed as a dendrogram. The dashed line clarifies the critical value of *d*_crit_ = 3.51, which corresponds to a significance level of 5%. The BACs are marked with numbers among the dendrogram and highlighted with the gray solid bar (technically correct BAC) and diagonally striped bar (faulty BAC).

**FIGURE 7:**
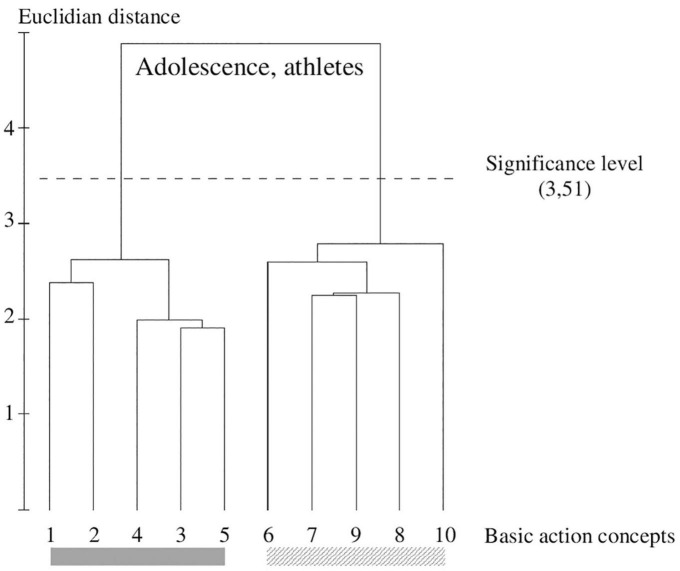
Outcome of the hierarchical cluster analysis of athletes within adolescents (*N* = 20) displayed as a dendrogram. The dashed line clarifies the critical value of *d*_crit_ = 3.51, which corresponds to a significance level of 5%. The BACs are marked with numbers among the dendrogram and highlighted with the gray solid bar (technically correct BAC) and diagonally striped bar (faulty BAC).

Figures 2–7 illustrate the average cognitive representation structure of all age groups and the level of expertise displayed as dendrograms. The BACs (pictures 1–5) marked with a gray solid bar are related to technically correct movements, and BACs (pictures 6–10) marked with a diagonally striped bar are related to technical faulty movements (pictures 6–10).

### Within-Group Comparison Novices Within Childhood

Within childhood, the group of novices (see Figure 2) shows four clusters in their representation structure. They clustered two technically correct (pictures 1 and 2; pictures 3–5) and two faulty (pictures 7–9; pictures 6 and 10) solutions.

### Within-Group Comparison of Novices Within Pubescence

In the cluster solution of novices within pubescence (see Figure 3), three clusters can be seen. In contrast to childhood, one technically correct cluster solution (pictures 1 and 2), one faulty solution (pictures 6, 8, and 10), and one mixed consisting of a correct and faulty solution (pictures 3, 5, 4, 7, and 9) were formed by novices. They could not separate the technically correct and faulty concepts from each other.

### Within-Group Comparison of Novices Within Adolescents

The cluster solution of novices within adolescents (see Figure 4) shows a similar structure to the novices within childhood. They indicate four clusters, two technically correct (pictures 1, 2, and 4; pictures 3 and 5) and two technically faulty (pictures 7, 9, and 8; pictures 6 and 10) solutions.

### Within-Group Comparison of Athletes Within Childhood

A similar cluster solution to the novice group within childhood is shown by athlete within childhood (Figure 5). They also separate two technical correct (pictures 1 and 2; pictures 3, 4, and 5) and two faulty (pictures 7, 9, and 8; pictures 6 and 10) clusters in their representation structure. For all four groups, the nodes are located slightly below the critical distance (*d*_crit_ = 3.51, *p* = 5%) and are only faintly connected to each other.

### Group of Athletes Within Pubescence and Adolescents

The cluster analysis (refer to Figures 6, 7) shows the representation structure for athletes in pubescence and adolescents. Their dendrograms clarify identical cluster solutions and show a clear separation in technically correct (pictures 1–5) and faulty BACs (pictures 6–10) as the RG. The hubs are highly interconnected and are clearly below the critical distance (*d*_crit_ = 3.51).

### Between-Group Comparisons

The invariance analysis indicates that the representation structures of overhead throwing movement were identical (λ = 1; λ_crit_ = 0.68) among these groups (refer to Table 2). The results highlighted that the novices in adolescents were at the level of the athletes within childhood. Table 2 clarifies that the representation structure for all age groups of novices and the athletes within childhood show no similarity (λ = 0.59; λ_crit_ = 0.68) with the representation structure of athletes in pubescence and adolescents. The cluster solutions also differ (λ = 0.59; λ_crit_ = 0.68) from the cluster solution of the RG, too (see Table 2). The dendrograms for athletes in pubescence and adolescents clarify identical cluster solutions as the RG. These cluster solutions match to RG (λ = 1; λ_crit_ = 0.68). Both group cluster solutions show two clusters and a clear separation in a technically correct (pictures 1–5) and faulty concepts (pictures 6–10). Similar to the RG, the nodes are highly interconnected and below the critical distance (*d*_crit_ = 3.51).

**TABLE 2:** Results (λ-values) of group analysis for cluster sets of each group.

Group	NC	NP	NA	HC	HP	HA	RG
NC		1	1	1	0.59	0.59	0.59
NP	1		1	1	0.59	0.59	0.59
NA	1	1		1	0.59	0.59	0.59
HC	1	1	1		0.59	0.59	0.59
HP	0.59	0.59	0.59	0.59		1	1
HA	0.59	0.59	0.59	0.59	1		1
RG	0.59	0.59	0.59	0.59	1	1	

*Outcome of invariance analysis between the mental structures of each group. NC, novices group within childhood; NP, novices group within pubescence; NA, novices group within adolescents; HC, handball group within childhood; HP, handball group within pubescence; HA, handball group within adolescents; RG, reference group, λ_crit_ = 0.68.*

## Discussion

The aim of this study was to explore age- and expertise-related differences in mental representation of the overhead throwing movement. The SDA-M (Schack, 2010) was used to analyze the mental representation.

All novices, children, pubescence, and adolescents showed the same unstructured mental representation, and there were no differences in the quality of the mental representation of the overhead throwing movement characteristics between the various levels of development in motor ontogenesis. All age groups could not distinguish the technically correct and faulty concepts from each other. Thus, the mental representation of overhead throwing movements did not develop as a function of age until adolescents. It is remarkable that children from 6 to 16 years remained on one level of development in the mental representation of the overhead throwing movement, which contradicts studies of De Waelle et al. (2021), Caeyenberghs et al. (2009), and Souto et al. (2020) who found age-related changes in the cognitive development.

Athletes up to the age of 12 years also demonstrated less structured mental representations. Clearly recognized is the development between childhood and pubescence of athletes. From the age of 13 years, athletes successfully separated technically correct and technically faulty overhead throwing movements. These representation structures were comparable with the ideal reference-cluster solution.

These results suggest an influence of cognitive improvement to movement development, which is consistent with the benefits in quantitative and qualitative movement characteristics. As hypothesized in this study, a growing expertise in throwing performance is accompanied by a development of the mental representation (Frank et al., 2016). With an increasing training level, children and adolescents showed a well-structured mental representation, that is, separating technically correct and technically faulty clusters. This outcome is in accordance with studies of Stoeckel et al. (2012) and Schack and Mechsner (2006). These results suggest that an improvement of the mental representation of the overhead throwing movement is accompanied by improvements in quantitative and qualitative characteristics (Gromeier et al., 2017, 2019). Hence, good performances in the quality and accuracy of movements are associated with a well-structured mental representation, that is, a clear separation in a technically correct and a technically faulty cluster. This confirms (Schmitz and Assaiante, 2002) in the way that a longer practice time develops an internal representation.

In contrast, the development of cognitive competences in overhead throwing movements is not linked to advancing age. It has been shown that between various levels of motor development (i.e., childhood, pubescence, and adolescents), no improvement could be recognized, and the novices stagnated on a low level of mental representation. Therefore, with increasing age, novices did not improve their mental representation as well as the quantitative and qualitative throwing performances (Gromeier et al., 2019). In sum, without an increased training, neither the throwing performance nor the associated mental representation is likely to improve further by itself or automatically. On the one hand, these findings contradict previous studies that found age-related differences in the development of the overhead throwing movement (Roberton and Langendorfer, 1980; Halverson et al., 1982; Winter, 1987; Roberton and Konczak, 2001; Lorson et al., 2013). In summary, these differences were found in throwing velocity, throwing accuracy, and movement quality characteristics. On the other hand, these findings underline recent research on the development of mental representation structure, which has elicited functional changes in novices’ representations as a result of physical and mental practice (Frank et al., 2014; Schack and Frank, 2021).

For physical education teachers and university sport lecturers, the results of this study imply that the development of complex motor skills, such as throwing movements, is not completed at the end of adolescents. Without specific interventions (e.g., technique practice), children and adolescents cannot evolve their cognitive and motor competences in throwing (Gromeier et al., 2019).

A reason can be found in the complexity of the overhead throwing movement. In learning processes of complex motor skills, children, youth, and adults are repeatedly confronted with excessive demands because of coordinative requirements (Hamilton and Tate, 2002). The optimal performance of overhead throwing movements requires precise mechanics that involve optimal execution of proximal-to-distal sequences to develop, transfer, and regulate the forces that are needed to withstand the inherent demands of the task (Kibler et al., 2013). To produce optimal speed and/or high accuracy at the distal end of the throwing arm, involved body segments have to be ordered in a specific way. The quality of overhead throwing movements highly depends of the object of throwing. In general, during the learning process of overhead throwing movements, educators must be ensured that the throwing object can be grasped with fingers and held securely with one hand. Regarding the physical education and university education, exercises should be applied purposefully, including individual instructions. To support the children in their motor development, the learning process should be supplemented by cognitive training to develop the mental representation of overhead throwing movements. To develop mental representation of a complex motor skill like the overhead throwing movement, there is evidence that physical education and training intervention should focus on random training (Fazeli et al., 2017). By comparing block and random practice, random training is more effective and could be beneficial in the transfer of learning to novel tasks (Jamieson and Rogers, 2000). The results suggested by Kim et al. (2017) supports the idea that being able to observe a new skill (e.g., throwing a ball) increases a person’s ability to implement that motor skill. Cognitive-perceptual and performance changes were improved over time through to action observation training (Kim et al., 2017).

These outcomes can only be interpreted within the limits of a cross-sectional study, which allows only restricted statements on the development. Due to the young participants, it was necessary to simplify and adapt the SDA-M (i.e., a group of faulty BACs, and a group of correct BACs), and the generalizability of these findings is subject to certain limitations. Further studies should examine differences and similarities in the mental representation of the overhead throwing movement of different age-bands and expertise, comparing with qualitative, quantitative, and biomechanical data. To examine difficulties in movement pattern among novices, further studies should evaluate the qualitative development across time of single movement characteristics and effects of specific cognitive training interventions on movement pattern. Concerning athletes, future studies should investigate if the similarities and differences in the athletes’ cognitive structures are also reflected in their motor performance. For both novices and experienced athletes, this can obtain further information on how to improve specific motions as well as on how to support an overall motor learning.

## Data Availability Statement

The raw data presented in this study are available upon reasonable request from the corresponding author (MG).

## Ethics Statement

The studies involving human participants were reviewed and approved by the Ethics Committee (”Ethik-Kommission”) at Bielefeld University. Written informed consent to participate in this study was provided by the participants’ legal guardian/next of kin.

## Author Contributions

MG: conception or design of the work, data collection, data analysis and interpretation, critical revision of the manuscript, and drafting the manuscript. TS: critical revision of the manuscript and final approval of the version to be published. DK: conception or design of the work, data analysis and interpretation, and critical revision of the manuscript. All authors contributed to the article and approved the submitted version.

## Conflict of Interest

The authors declare that the research was conducted in the absence of any commercial or financial relationships that could be construed as a potential conflict of interest.

## Publisher’s Note

All claims expressed in this article are solely those of the authors and do not necessarily represent those of their affiliated organizations, or those of the publisher, the editors and the reviewers. Any product that may be evaluated in this article, or claim that may be made by its manufacturer, is not guaranteed or endorsed by the publisher.
